# IL-10^+^ NK and TGF-β^+^ NK cells play negative regulatory roles in HIV infection

**DOI:** 10.1186/s12879-018-2991-2

**Published:** 2018-02-13

**Authors:** Yongjun Jiang, Mei Yang, Xiaojuan Sun, Xi Chen, Meichen Ma, Xiaowan Yin, Shi Qian, Zining Zhang, Yajing Fu, Jing Liu, Xiaoxu Han, Junjie Xu, Hong Shang

**Affiliations:** 1 Key Laboratory of AIDS Immunology of National Health and Family Planning Commission, Department of Laboratory Medicine, The First Affiliated Hospital, China Medical University, No. 155, Nanjingbei Street, Heping District, Shenyang, Liaoning Province 110001 China; 2 Collaborative Innovation Center for Diagnosis and Treatment of Infectious Diseases, 79 Qingchun Street, Hangzhou, China; 3 Clinical Laboratory, Shenyang Women and Children’s Hospital, Shenyang, China

**Keywords:** HIV, IL-10, TGF-β, NK, Antiretroviral treatment, IFN-γ, Immune regulation

## Abstract

**Background:**

Natural killer (NK) cells play cytotoxic roles by targeting tumor cells or virus infected cells, they also play regulatory roles by secreting cytokines and chemokines. Transforming growth factor (TGF)-β and interleukin (IL)-10 are important immunosuppressive cytokines potentially related to the immune dysregulation that occurs in the infection of human immunodeficiency virus (HIV). NK cells are an important source of TGF-β and a main early producer of IL-10 in response to viral infection. Here, we evaluated the percentages of IL-10^+^ and TGF-β^+^ NK cells in HIV-infected patients relative to healthy controls (HCs).

**Methods:**

Study participants (*n* = 63) included 31 antiretroviral treatment (ART)-naïve HIV-infected patients, 17 ART-treated HIV-infected patients, and 15 HIV-negative HCs. Expression of IL-10 or TGF-β in NK cells was examined by flow cytometry, and the influences of recombinant IL-10 (rIL-10) or recombinant TGF-β (rTGF-β) on NK cell function were investigated in vitro.

**Results:**

Compared with HCs, ART-naïve HIV-infected patients had increased percentages of IL-10^+^ (2.0% vs. 0.4%, *p* = 0.015) and TGF-β^+^ (4.5% vs. 2.1%, *p* = 0.022) NK cells, and ART-treated patients also had a higher percentage of IL-10^+^ NK cells (2.5% vs. 0.4%, *p* = 0.002). The percentages of IL-10^+^ and TGF-β^+^ NK cells were positively correlated (r = 0.388; *p* = 0.010). The results of in vitro experiments demonstrated that rIL-10 and rTGF-β inhibited NK cell CD107a expression (*p* = 0.037 and *p* = 0.024, respectively), IFN-γ secretion (*p* = 0.006, *p* = 0.016, respectively), and granzyme B release after stimulation (*p* = 0.014, *p* = 0.040, respectively).

**Conclusions:**

Our data suggest that the percentages of IL-10^+^ or TGF-β^+^ NK cells are increased in HIV-infected patients, and that rIL-10 and/or rTGF-β can inhibit NK cell functions in vitro, providing a potential therapeutic target for strategies aimed at combating HIV infection.

## Background

Natural killer (NK) cells serve as the first line of immune defense in host protection against viruses and tumors [1]. In humans, NK cells account for 2%–18% of the lymphocytes in peripheral blood and express various inhibitory and activating receptors, including C-type lectin-like, natural cytotoxicity, and killer cell immunoglobulin-like receptors [2, 3]. NK cell functions include killing target cells, cytokine production, and antibody-dependent cellular cytotoxicity (ADCC) [2]. Moreover, NK cells are critical effectors mediating cytotoxicity, and regulators modulating the activation and development of other immune response components [1]. NK cells are identified via their lack of CD3 and expression of CD56 cell surface markers, and they can be further divided into CD56^dim^ and CD56^bright^ subsets [3]. Generally, CD56^dim^ NK cells release perforin or granzymes, which play a key role in killing target cells, whereas CD56^bright^ NK cells secrete interleukin (IL)-10, interferon (IFN)-γ, transforming growth factor (TGF)-β and other cytokines, to exert immunomodulatory effects [4–6].

IL-10 and TGF-β are important immunoregulatory cytokines in vivo [7, 8], which suppress adaptive and innate immunity [9]. IL-10 is produced by multiple cell types, including T cells, NK cells, monocytes, and B cells; NK cells are a major early source of this cytokine in response to viral infection [10–13]. IL-10 is involved in the impairment of T cell function during persistent viral infections, and blockage of the IL-10 pathway alone is sufficient to restore T cell activities and increase viral control [14]. TGF-β is also secreted by various cell types, particularly NK cells, which are the only lymphocyte population that constitutively produces this cytokine [15]. TGF-β plays important roles in immunomodulation, inflammation, and tissue repair [16], and can inhibit T cell proliferation and cytotoxicity [17]. IL-10 is reported to cause harmful effects during human immunodeficiency virus (HIV) infection by reducing IL-2 and IL-12 production, thereby inhibiting antigen-presentation and cellular immune responses [18–20]. HIV-infected CD4^+^ T cells can produce IL-10, leading to persistent viral infection [11]. High levels of TGF-β in the plasma were reported in HIV-infected patients compared with healthy controls (HCs) [21]; however, the cell types producing TGF-β in this context remain to be determined.

IL-10^+^ NK cells play significant modulatory roles in various viral, bacterial, and parasitic infections [12, 22–24]. TGF-β^+^ NK cells have been reported to serve as an important co-stimulatory signal to induce suppressive T cells [15]. In HIV infection, multiple cells can produce IL-10 and TGF-β. The majority of research has focused only on T cells, rather than NK cells, which are a major source of these cytokines and play important roles during acute HIV infection. The percentage of IL-10^+^ or TGF-β^+^ NK cells in HIV-infected patients and the regulatory effect of IL-10 and TGF-β have yet to be elucidated.

In the present study, we determined the percentages of IL-10^+^ and TGF-β^+^ NK cells in HIV-infected patients and healthy controls (HCs). We also explored the immunomodulatory effects of recombinant IL-10 (rIL-10) and recombinant TGF-β (rTGF-β) on NK cell functions, including the expression of lysosomal-associated membrane glycoprotein-1 (LAMP1; also known as CD107a), and IFN-γ secretion. The results indicated that IL-10^+^ and TGF-β^+^ NK cells may be risk factors for HIV disease progression, and are potential therapeutic targets in combating HIV infection.

## Methods

### Study participants

Sixty-three individuals participated in this study, including 31 antiretroviral treatment-naïve HIV-infected patients (ART-naïve), 17 ART-treated HIV-infected patients (ART-treated), and 15 HIV-negative HCs. The 31 ART-naïve patients had a median CD4^+^ T cell count of 363 cells/μl (interquartile range [IQR] 277–421 cells/μl) and median viral load of 24,760 copies/ml (range, 374–388,000 copies/ml). ART-naïve patients were further grouped according to CD4^+^ T cell counts (CD4^+^ T ≥ or < 350 cells/μl groups) or viral load (viral load > 10^4^ or ≤10^4^ copies/ml groups). The 17 ART-treated patients had received ART for ≥2 years, and had undetectable viral loads (HIV RNA, < 20 copies/ml); the median duration of their virologic suppression at the time of sampling was 33 months (IQR: 24–60 months), and they had a median CD4^+^ T cell count of 586 cells/μl (IQR: 453–684 cells/μl). HCs were HIV-negative, anti-hepatitis C antibody-negative, and hepatitis B surface antigen-negative, with normal liver and kidney functions, and without immune-system diseases.

### Detection of IL-10^+^ or TGF-β^+^ NK cells by flow cytometry

**Fig. 1:**
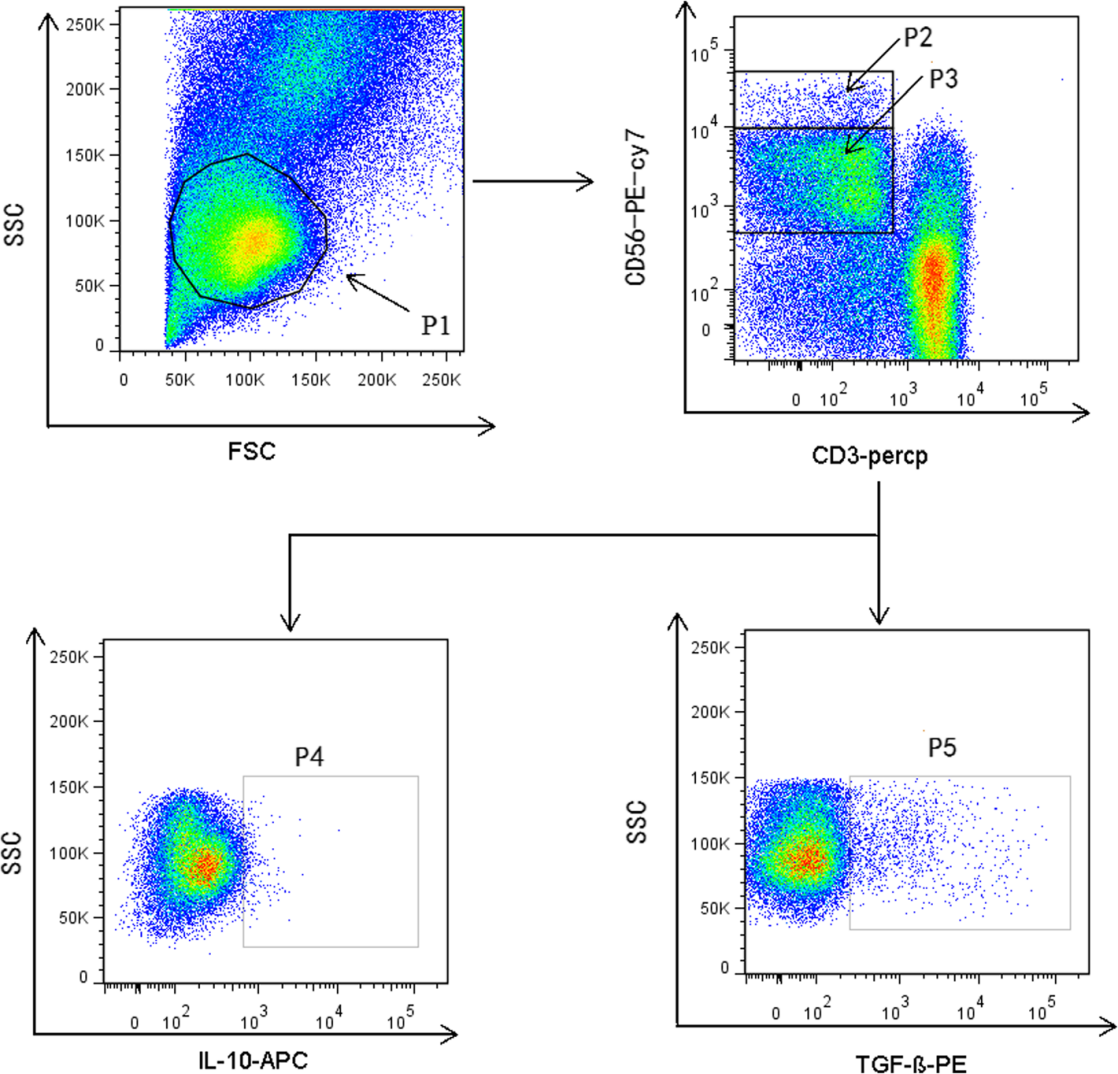
Gating strategy for identification of NK cells and their subsets P1 represents lymphocytes identified using a side and forward scatter dot plot. P2 represents CD56^bright^ NK cells; gating, CD3^−^ CD56^bright^. P3 represents CD56^dim^ NK cells; gating, CD3^−^ CD56^dim^. P2 + P3 represents total NK cells. P4 represents IL-10^+^ NK cells. P5 represents TGF-β^+^ NK cells

Peripheral blood mononuclear cells (PBMCs) were isolated from HIV-infected patients and HCs by Ficoll™ density gradient centrifugation. PBMCs were cultured for 6 h with recombinant IL-12 (rIL-12, 10 ng/ml) and recombinant IL-15 (rIL-15, 50 ng/ml) in a 5% CO_2_ incubator at 37 °C. Total NK cells and NK cell subsets were defined by their expression of CD3 and CD56, detected using the anti-CD3 PerCP and anti-CD56 PE-Cy7 antibodies (BD Biosciences, San Jose, CA, USA), respectively. Cells were stained with intracellular anti-TGF-β-PE and anti-IL-10-APC (Biolegend, San Diego, CA, USA) and fixed in 1% paraformaldehyde before detection using a flow cytometer (LSR II; Becton Dickinson, Franklin Lakes, NJ, USA) and analysis with FACSDiva™ software (Becton Dickinson); the gating strategy is shown in Fig. 1.

### Determination of NK cell CD107a expression and IFN-γ secretion

PBMCs were co-cultured with different concentrations of rIL-10 and/or rTGF-β for 5 h, then stimulated with rIL-12 (10 ng/ml, R&D) and rIL-15 (50 ng/ml, R&D) for 24 h at 37 °C, and anti-CD107a-PE antibody (Biolegend, San Diego, CA, USA) added simultaneously. Cells were co-cultured in GolgiStop™ ((Becton Dickinson) for ≥5 h before the end of stimulation. PBMCs were collected, and anti-CD3-Percp and anti-CD56-PE-cy7 were used for surface staining. Cells were then stained for intracellular IFN-γ with anti-IFN-γ-APC antibody (BD Biosciences, San Jose, CA, USA), washed with phosphate-buffered saline, and fixed in 1% paraformaldehyde, followed by analysis using the LSR II flow cytometer.

### Determination of NK cell release of granzyme B and perforin

PBMCs were co-cultured with different concentrations of rIL-10 and/or rTGF-β for 5 h. Then, cells were stimulated with PMA (10 ng/μL) and ionomycin (1 μg/μL) (Sigma Chemical St. Louis, MO, USA) for 6 h at 37 °C. PBMCs were collected, and surface-stained with anti-CD3-PerCP and anti-CD56-PE-Cy7. Anti-granzyme B-PE and anti-perforin-fluorescein isothiocyanate (FITC) (BD Biosciences, San Jose, CA, USA) were used for intracellular staining, and cells detected by flow cytometry (LSR II).

### Detection of CD4^+^T cell count

CD4^+^ T cell counts were determined using a flow cytometer (FACS Calibur; Becton Dickinson). High, medium, and low Trucount™ Control Beads (BD Biosciences, San Jose, CA, USA) were used to confirm the accuracy and quality of CD4^+^ T cell counts. A single-platform lyse-no-wash procedure was conducted with TriTEST™ anti-CD3-PerCP/CD4-FITC/CD8-PE reagents and Trucount tubes (BD Biosciences, San Jose, CA, USA) according to the manufacturer’s instructions.

### Determination of HIV viral load

Plasma HIV RNA levels were determined with by reverse transcription-polymerase chain reaction (RT-PCR) using the COBAS®AmpliPrep®/COBAS TaqMan™ HIV test v2.0 (Roche Diagnostic Systems, Basel, Switzerland). The detection range of this method is 20–10,000,000 copies/ml. The number of HIV RNA copies was calculated according to the manufacturer’s reference standard.

### Statistical analyses

The non-parametric Mann–Whitney *U*-test was used for evaluation of differences between two groups, and the Kruskall–Wallis test employed for comparisons of multiple groups. The Friedman’s test was used for multiple pairwise comparisons. Correlation analysis was performed using the Spearman’s rank correlation test. All analyses were undertaken using SPSS v19.0 (IBM, Armonk, NY, USA). *p* < 0.05 was considered significant.

## Results

### Percentages of total NK cells and NK subsets in HIV-infected patients and HCs

The clinical characteristics of ART-naïve and ART-treated patients, and HCs, are presented in Table 1. The sex and age of HIV-infected patients were matched with those of HCs. The percentages of total NK cells and NK subsets among lymphocytes in peripheral blood samples from the three groups were measured by flow cytometry.

**Table 1:** Clinical characteristics of study subjects

Parameters	HC(*n* = 15)	ART-naive patients(*n* = 31)	ART-treated patients(*n* = 17)
	Median(IQR)	Median(IQR)	Median(IQR)
Sex	male	male	male
Age(years)	27 (25–33)	25 (21–37)	30 (27.5–38)
CD4^+^T cells (cells/μl)	ND	363 (277–421)	586 (453–684)
HIV Viral Load(copies/ml) Log10	NA	4.19 (3.87–4.86)	Undetectable (< 1.30)
Months since infection	NA	33 (19–68)	67 (40.5–86.5)
Months of ART treatment	NA	NA	42 (30–66)

NOTE: ND: no data; NA: not applicable

**Fig. 2:**
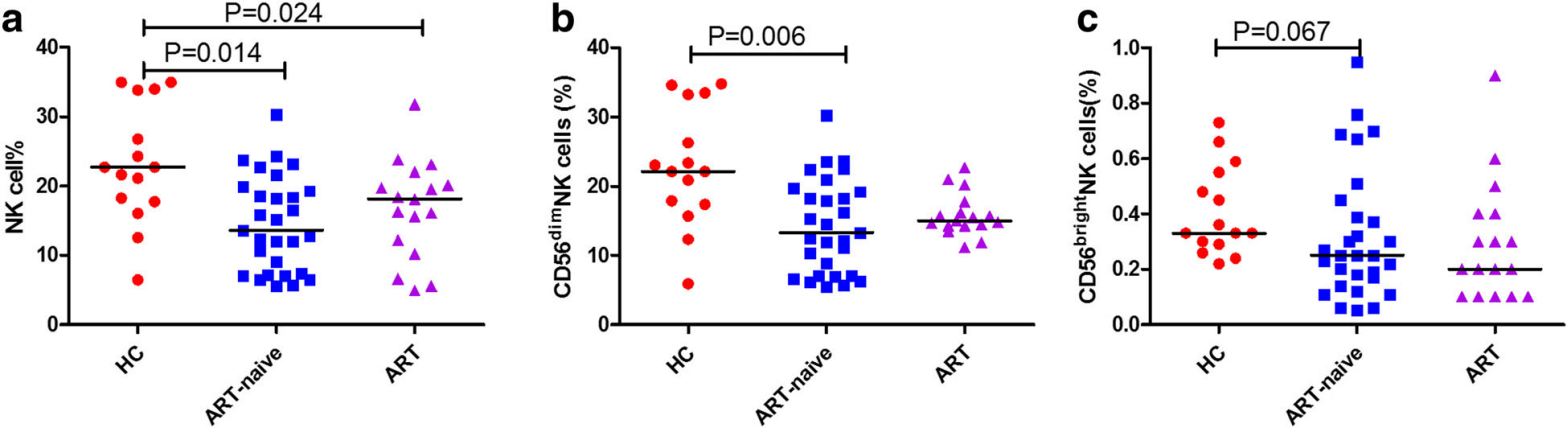
Percentages of total NK cells and NK cell subsets in HIV-infected patients and HCs The percentages of total NK cells among lymphocytes from HCs, ART-naïve, and ART-treated patients (**a**). The percentages of NK cell subsets (CD56^dim^ and CD56^bright^) in lymphocytes from HCs, ART-naïve, and ART-treated patients (**b**, **c**)

The percentages of total NK cells in ART-naïve and ART-treated patient samples were lower than that in HCs (*p* = 0.014, *p* = 0.024) (Fig. 2a). In addition, the percentages of CD56^dim^ NK cells in ART-naïve patients were lower than those in HCs (*p* = 0.006) (Fig. 2b) and there was a tendency towards lower percentages of CD56^bright^ NK cells in ART-naïve patients compared with those of HCs (*p* = 0.067) (Fig. 2c).

### Percentages of IL-10^+^ or TGF-β^+^ NK cells in HIV-infected patients

**Fig. 3:**
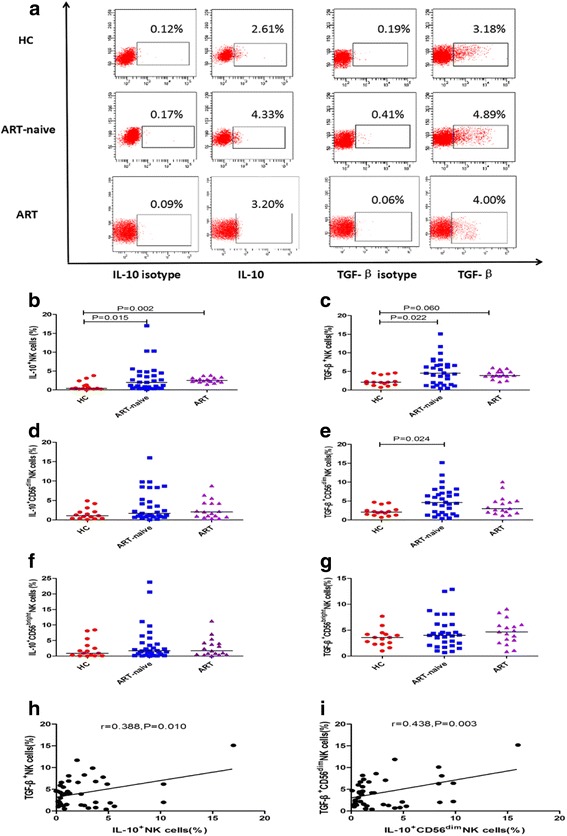
Comparison of the percentages of IL-10^+^ and TGF-β^+^ NK cells in HIV-infected patients and HCs PBMCs from HCs, ART- naïve, and ART-treated patients were stimulated using rIL-12 (10 ng/ml) and rIL-15 (50 ng/ml). IL-10^+^ and TGF-β^+^ NK cells were examined by multi-color flow cytometry, with gates defined according to isotype controls. Representative flow-cytometric plots of IL-10^+^ and TGF-β^+^ NK cells in HIV-infected patients and HCs (**a**). Comparison of the percentages of IL-10^+^ NK cells (**b**), TGF-β^+^ NK cells (**c**), IL-10^+^ CD56^dim^ NK cells (**d**), TGF-β^+^ CD56^dim^ NK cells (**e**), IL-10^+^ CD56^bright^ NK cells (**f**), and TGF-β^+^ CD56^bright^ NK cells (**g**), among the three groups. Correlation between the percentage of IL-10^+^ NK cells and TGF-β^+^ NK cells (**h**). Correlation between the percentage of IL-10^+^ CD56^dim^ NK cells and TGF-β^+^ CD56^dim^ NK cells (**i**)

PBMCs obtained from HCs, and ART-naïve, and ART-treated patients, were stimulated with rIL-12 and rIL-15. The percentage of IL-10^+^ or TGF-β^+^ NK cells was examined by intracellular staining and multi-color flow cytometry. Representative flow cytometry plots of IL-10 or TGF-β expression in NK cells from each participant group are shown in Fig. 3a. The percentage of IL-10^+^ NK cells in ART-naïve patients was higher than that in HCs (*p* = 0.015); moreover, the percentage of IL-10^+^ NK cells was also higher in the ART-treated HIV patient group than that in HCs (*p* = 0.002) (Fig. 3b). The percentage of TGF-β^+^ NK cells in ART-naïve patients was elevated compared with that of HCs (*p* = 0.022), while in ART-treated patients, the percentage of TGF-β^+^ NK cells still exhibited a tendency to be higher than that of HCs (*p* = 0.060) (Fig. 3c). There was no difference in the percentage of IL-10^+^ CD56^dim^ NK cells among the three groups (Fig. 3d), whereas the percentage of TGF-β^+^ CD56^dim^ NK cells in ART-naïve patients was higher than that in HCs (*p* = 0.024) (Fig. 3e). Moreover, there were no differences in the percentages of IL-10^+^ CD56^bright^ and TGF-β^+^ CD56^bright^ NK cells among the three groups (Fig. 3f, g). In ART-naïve patients, the percentage of IL-10^+^ NK cells was positively correlated with that of TGF-β^+^ NK cells (r = 0.388, *p* = 0.010) (Fig. 3h) and the percentage of IL-10^+^ CD56^dim^ NK cells correlated positively with that of TGF-β^+^ CD56^dim^ NK cells (r = 0.438, *p* = 0.003) (Fig. 3i).

### Comparison of the percentages of IL-10^+^ and TGF-β^+^ NK cells in different groups according to CD4^+^ T cell count or HIV viral load

**Fig. 4:**
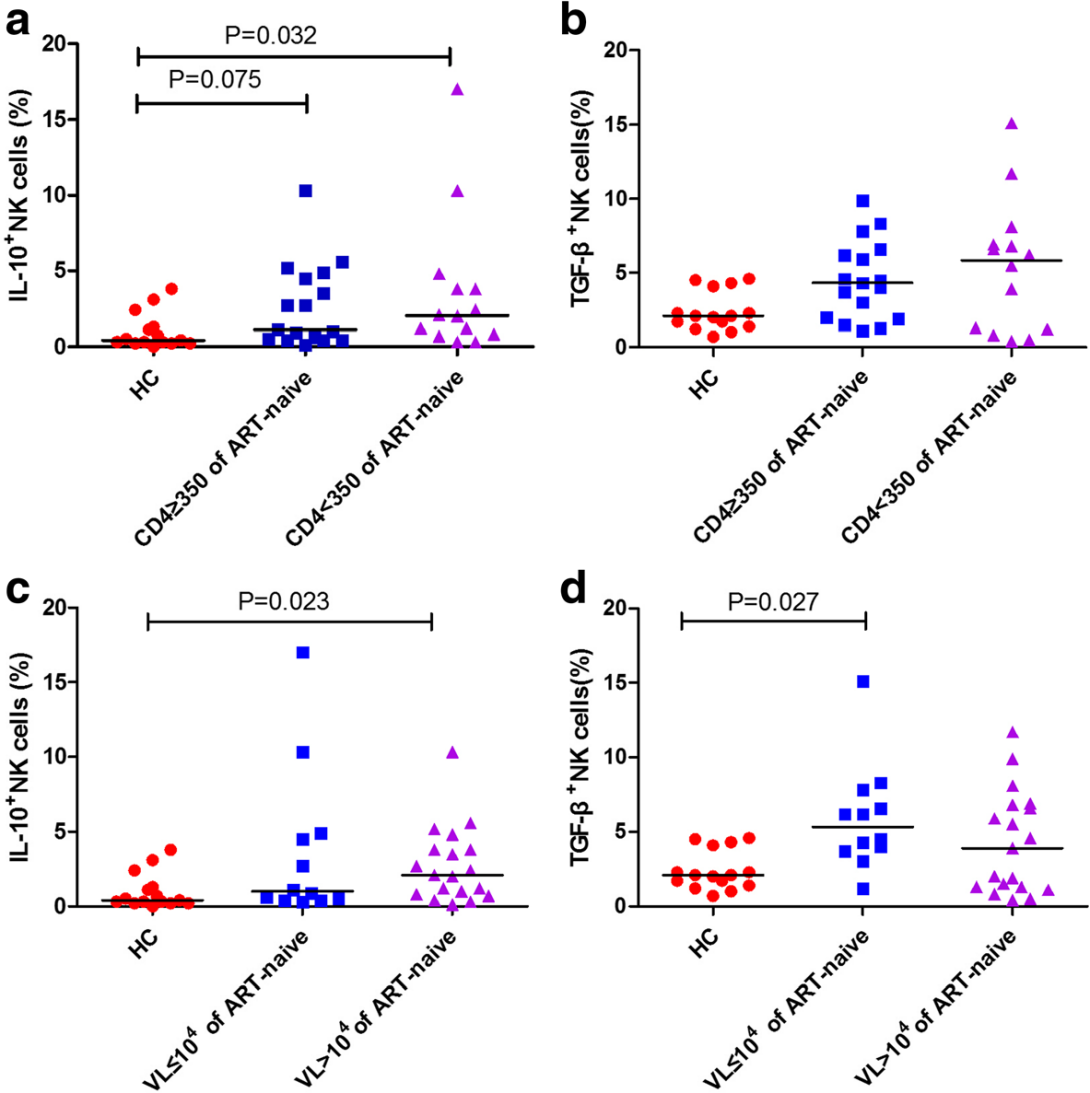
Percentage of IL-10^+^ or TGF-β^+^ NK cells in different groups The percentage of IL-10^+^ or TGF-β^+^ NK cells in different groups according to CD4^+^ T cell counts (**a**, **b**). The percentage of IL-10^+^ or TGF-β^+^ NK cells in different groups according to viral load (**c, d**)

CD4^+^ T cell counts and viral loads are important markers for HIV disease progression. We analyzed the correlation between the percentage of IL-10^+^ or TGF-β^+^ NK cells and CD4^+^ T cell counts and viral loads and found no significant correlations between them. Furthermore, we analyzed groups subdivided by CD4^+^ T cell count or viral load. The percentage of IL-10^+^ NK cells in the CD4^+^ T < 350 cells/μl group of ART-naïve patients was higher than that of the HC group (*p* = 0.032), and there was an increasing trend in the CD4^+^ T ≥ 350 cells/μl group of ART-naïve patients compared with HCs (*p* = 0.075) (Fig. 4a). There was no difference in the percentages of TGF-β^+^ NK cells among groups (Fig. 4b). When grouped according to the level of viral load, the percentage of IL-10^+^ NK cells in ART-naïve patients with viral load > 10^4^ copies/ml was higher than that in the HC group (*p* = 0.023) (Fig. 4c), and that in the group with viral load < 10^4^ copies/ml, while the percentage of TGF-β^+^ NK cells was higher than that in the HC group (*p* = 0.027) (Fig. 4d).

### Effect of rIL-10 and rTGF-β on the NK cell CD107a expression and IFN-γ secretion

**Fig. 5:**
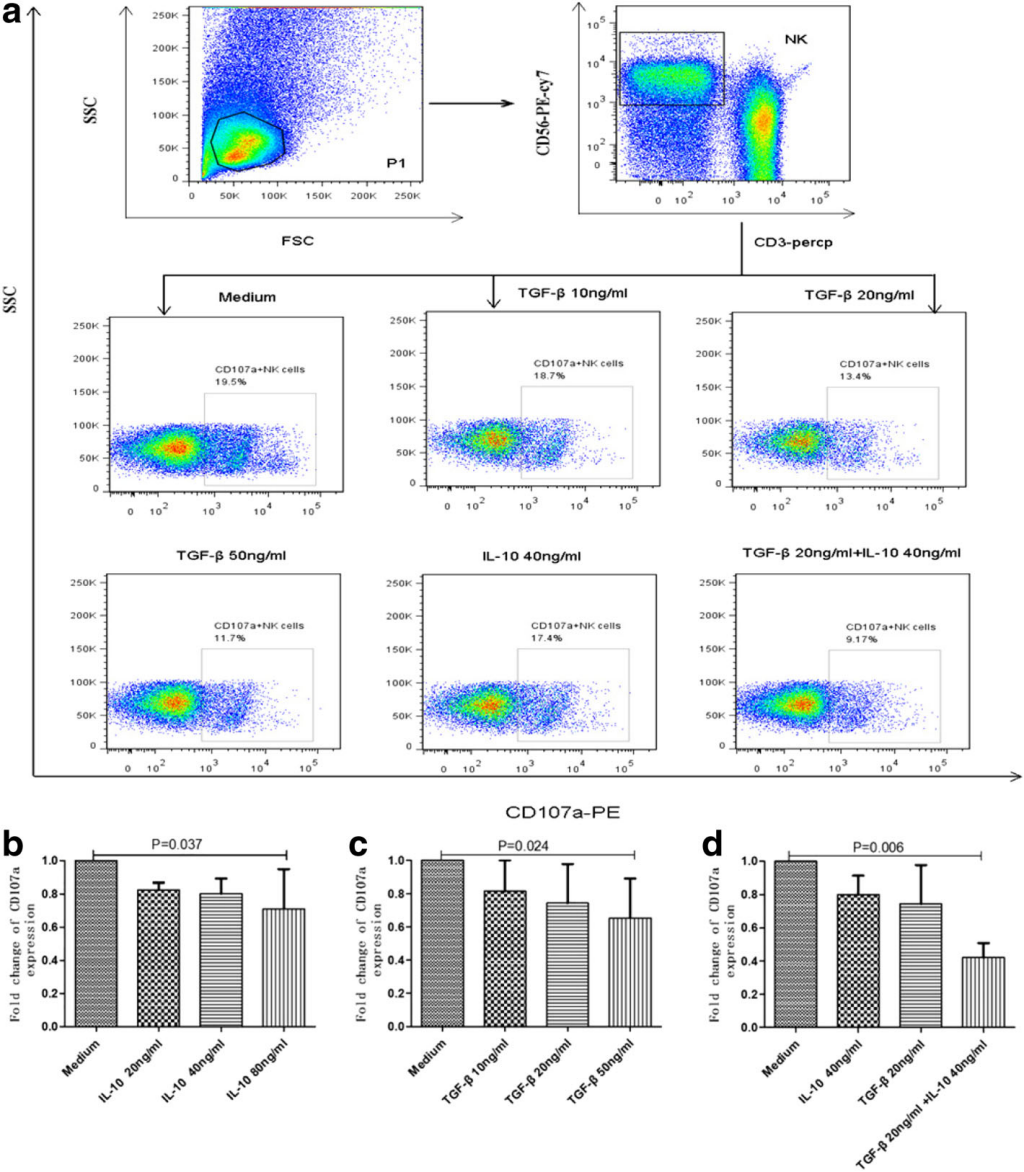
Effect of rIL-10 and rTGF-β on CD107a expression in NK cells PBMCs were incubated with rIL-10 or rTGF-β and CD107a expression by NK cells evaluated. Representative flow-cytometry plots demonstrated the effect of rIL-10 or rTGF-β on CD107a expression in NK cells (**a**). CD107a expression by NK cells was inhibited by different concentrations of rIL-10 (**b**) or rTGF-β (**c**). Fold-change in CD107a expression by NK cells was synergistically inhibited by rIL-10 and rTGF-β (**d**). Fold-change of CD107a expression = (percentage CD107a expression in untreated controls − percentage of CD107a expression after treatment with rIL-10 and/or rTGF-β)/percentage of CD107a expression in untreated controls

**Fig. 6:**
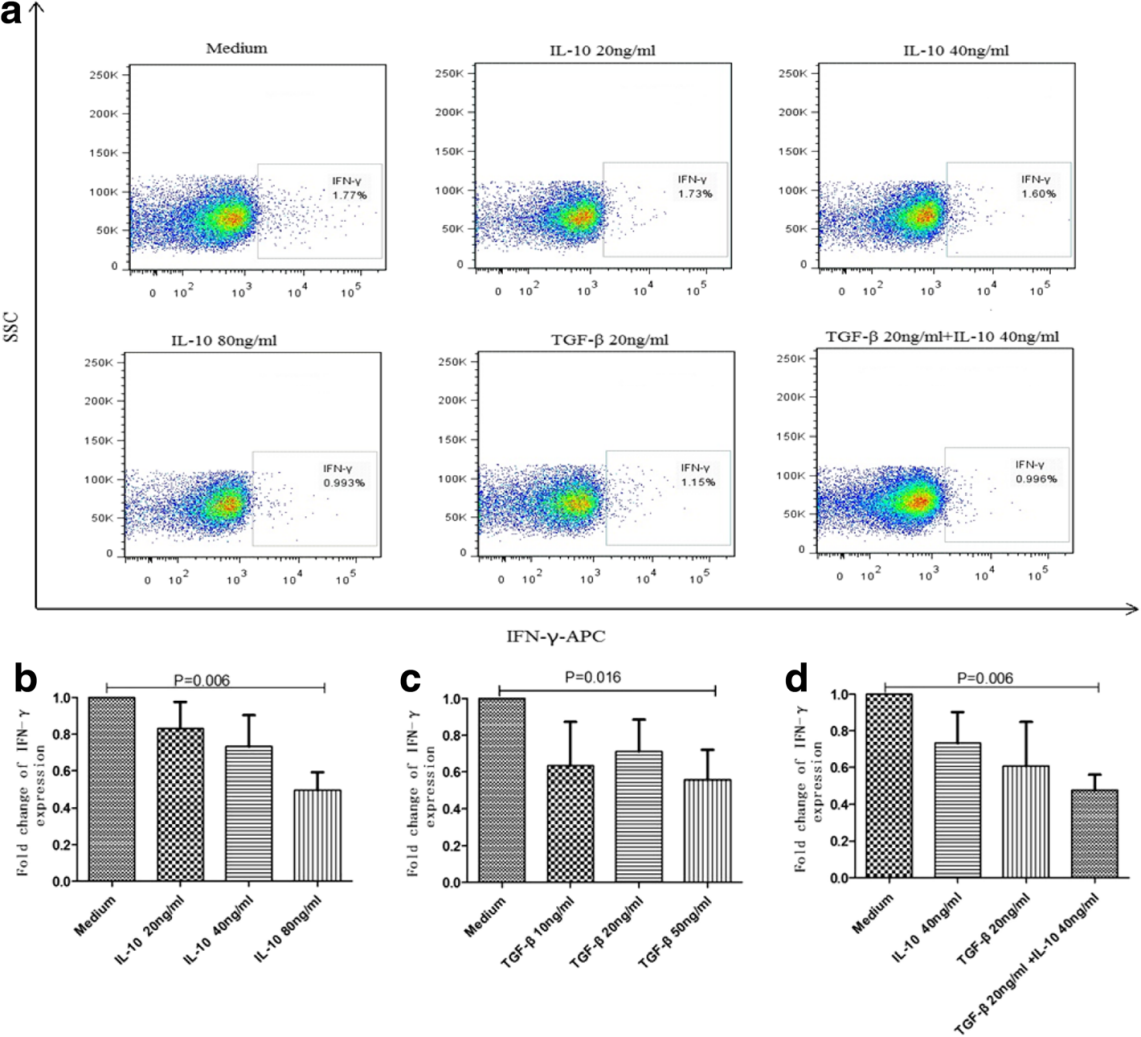
Effect of rIL-10 and rTGF-β on IFN-γ secretion by NK cells PBMCs were co-cultured with rIL-10 and/or rTGF-β for 5 h, then stimulated with rIL-12 and rIL-15 for 24 h. Representative flow-cytometry plots demonstrated the effect of rIL-10 or rTGF-β on IFN-γ secretion by NK cells (**a**). IFN-γ secretion by NK cells was inhibited by different concentrations of rIL-10 (**b**) or rTGF-β (**c**) and synergistically by treatment with both rIL-10 and rTGF-β (**d**)

The expression of CD107a and IFN-γ secretion can be used to assess the functional anti-viral ability of NK cells. The inhibitory effects of rIL-10 or rTGF-β on NK cell functions were evaluated, and the percentages of IFN-γ^+^ and CD107a^+^ NK cells determined. Representative flow cytometry plots, demonstrating the effects of rIL-10 or rTGF-β on CD107a expression by NK cells are presented in Fig. 5a. CD107a expression in NK cells was inhibited by high concentrations of rIL-10 or rTGF-β in vitro (*p* = 0.037, *p* = 0.024), and the rate of inhibition increased gradually with increasing concentrations of these cytokines (Fig. 5b, c). Moreover, the effect of rIL-10 and rTGF-β on CD107a expression in NK cells was synergistic, demonstrating inhibitory effects much greater than those of either cytokine individually (*p* = 0.006) (Fig. 5d). Representative flow-cytometric plots demonstrating the effects of rIL-10 or rTGF-β on IFN-γ secretion from NK cells are shown in Fig. 6a. IFN-γ secretion by NK cells was also inhibited by rIL-10 or rTGF-β (*p* = 0.006, *p* = 0.016), and the rate of inhibition increased gradually with their concentrations (Fig. 6b, c). Again, the effect of both IL-10 and TGF-β was synergistic (*p* = 0.006) (Fig. 6d).

### Effects of rIL-10 and rTGF-β on granzyme B and perforin release by NK cells

**Fig. 7:**
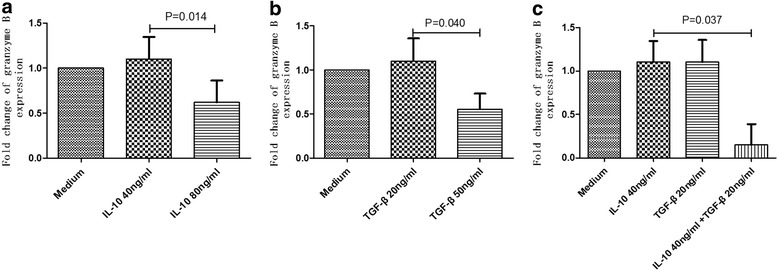
Effects of rIL-10 and rTGF-β on granzyme-B release by NK cells PBMCs were co-cultured with rIL-10 and/or rTGF-β for 5 h, and then stimulated with PMA and ionomycin for 6 h. The inhibition of granzyme-B release by NK cells using different concentrations of rIL-10 (**a**) or rTGF-β (**b**) was determined. The effect of treatment with both rIL-10 and rTGF-β on granzyme-B release by NK cells was synergistic (**c**)

**Fig. 8:**
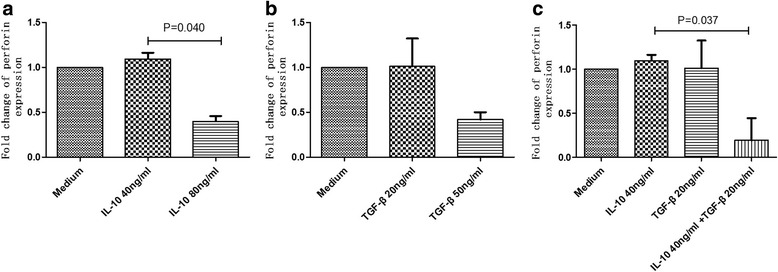
Effects of rIL-10 and rTGF-β on perforin release by NK cells PBMCs were co-cultured with rIL-10 and/or rTGF-β for 5 h, and then stimulated with PMA and ionomycin for 6 h. The inhibition of perforin release by NK cells by different concentrations of rIL-10 (**a**) or rTGF-β (**b**) was determined. The effects of treatment with both rIL-10 and rTGF-β on perforin release by NK cells were synergistic (**c**)

Granzyme B and perforin are important functional markers for NK cells, which indicate the killing capability of NK cells. The percentages of IL-10^+^ and TGF-β^**+**^ NK cells were elevated in HIV infected patients; however, their influence on the release of granzyme B and perforin by NK cells is not known. Our results demonstrate that a low concentration of rIL-10 or rTGF-β did not inhibit the release of granzyme B, whereas high concentrations of either cytokine did inhibit the release of this factor (*p* = 0.014, *p* = 0.040) (Fig. 7a, b). A synergistic effect of rIL-10 and rTGF-β on inhibition of the release of granzyme B was also noted (*p* = 0.037) (Fig. 7c). Similarly, rIL-10 and rTGF-β inhibited perforin release by NK cells (Fig. 8a–c).

## Discussion

Regulation of the immune system has attracted considerable attention over the last decade, and the cytokines, IL-10 and TGF-β, have important roles in controlling immune processes. The suppression of anti-cancer immunity by TGF-β and IL-10 has been reported in several studies [25–28]. In breast cancer, circulating levels of IL-10^+^ and TGF-β^+^ NK cells are increased, which inhibits the production of the NK cell effectors, IFN-γ and CD107a, and promotes the migration and invasion of breast-cancer cells [9].

Brockman et al. reported that IL-10 mRNA levels were increased in several PBMC subpopulations in HIV-infected patients compared with HCs, particularly T cells, B cells, monocytes, and NK cells, and that the inhibition of the IL-10 pathway enhanced the proliferative capacity of T cells [29]. Based on these findings regarding the levels of IL-10 mRNA in NK cells, we further explored the intracellular secretion of IL-10 and TGF-β proteins in NK cells (total and subsets) and investigated the relationship between IL-10^+^/TGF-β^+^ NK cells and CD4^+^ T cell counts/viral loads. Our data elucidate the effects of IL-10 and TGF-β on NK cell functions, including IFN-γ secretion, CD107a expression, and granzyme B or perforin release.

We found that ART-naïve patients had elevated percentages of IL-10^+^ NK cells relative to HCs; however, patients who had received ART, also had higher percentages of IL-10^+^ NK cells than those of HCs. Comparisons of TGF-β^+^ NK cell percentages among study groups generated similar results to those for IL-10^+^ NK cells. These data indicate that ART is insufficient to induce complete recovery of immune functions, and that specific immune therapies are required to overcome the negative regulation. Our previous study showed that HIV infection can result in impaired NK cell functions, including diminished levels of perforin and IFN-γ production [30, 31], which may be related to negative regulation by cytokines. In the present study, the results of our in vitro experiments demonstrate that the expression of CD107a and secretion of IFN-γ by NK cells can be inhibited by rIL-10 or rTGF-β. High concentrations of rIL-10 or rTGF-β could also inhibit the release of granzyme B and perforin; therefore, we suspect that the increase in the percentage of IL-10^+^ and TGF-β^+^ NK cells may be related to impaired NK cell cytotoxic function during HIV infection.

The mechanisms underlying the elevated percentage of IL-10^+^ and TGF-β^+^ NK cells during HIV infection remain unclear; however, it was reported that the HIV Env and Tat peptides are responsible for IL-10 production by CD4^+^ T cells [32–34], and the mitogen-activated protein kinase/extracellular signal-regulated kinase pathway was reported as involved in this process [35, 36]. Further, in vitro experiments have indicated that HIV Tat can induce TGF-β production by NK cells [21], and that TGF-β may suppress NK cell functions by repressing the mTOR pathway [37], or inhibit IFN-γ secretion and the ADCC response of NK cells via SMAD3-mediated signaling [38].

## Conclusions

The results of our study demonstrate that the percentages of IL-10^+^ and TGF-β^+^ NK cells are elevated in ART-naïve HIV-infected patients, and that ART-treated HIV patients also have a high percentage of IL-10^+^ NK cells. We also show that rIL-10 and/or rTGF-β can inhibit the functions of NK cell effectors. Our research provides a potential therapeutic target for combating HIV infection, along with information useful for research into the pathogenesis and immunotherapy of HIV infection.

## Abbreviations

ADCC: Antibody-dependent cellular cytotoxicity
ART: Antiretroviral treatment
HCs: Healthy controls
HIV: Human immunodeficiency virus
IFN: Interferon
IL: Interleukin
NK: Natural killer
PBMCs: Peripheral blood mononuclear cells
rIL: Recombinant interleukin
rTGF-β: Recombinant TGF-β
TGF-β: Transforming growth factor-β
